# Menopause and adipose tissue: miR-19a-3p is sensitive to hormonal replacement

**DOI:** 10.18632/oncotarget.23406

**Published:** 2017-12-18

**Authors:** Reeta Kangas, Cristina Morsiani, Grazia Pizza, Catia Lanzarini, Pauliina Aukee, Jaakko Kaprio, Sarianna Sipilä, Claudio Franceschi, Vuokko Kovanen, Eija K. Laakkonen, Miriam Capri

**Affiliations:** ^1^ Gerontology Research Center, Faculty of Sport and Health Sciences, University of Jyväskylä, Jyväskylä, Finland; ^2^ DIMES-Department of Experimental, Diagnostic and Specialty Medicine, University of Bologna, Bologna, Italy; ^3^ Epigenetics Program, Babraham Institute, Cambridge, United Kingdom; ^4^ Department of Obstetrics and Gynecology, Pelvic Floor Research and Therapy Unit, Central Finland Central Hospital, Jyväskylä, Finland; ^5^ Institute for Molecular Medicine Finland (FIMM) and Department of Public Health, University of Helsinki, Helsinki, Finland; ^6^ CIG- Interdepartmental Centre “Galvani”, Via Petronio Vecchi, University of Bologna, Bologna, Italy

**Keywords:** microRNAs, miR-19a-3p, adipose tissue, aging, estrogen therapy

## Abstract

Tissue-specific effects of 17β-estradiol are delivered via both estrogen receptors and microRNAs (miRs). Menopause is known to affect the whole-body fat distribution in women. This investigation aimed at identifying menopause- and hormone replacement therapy (HRT)-associated miR profiles and miR targets in subcutaneous abdominal adipose tissue and serum from the same women. A discovery phase using array technology was performed in 13 women, including monozygotic twin pairs discordant for HRT and premenopausal young controls. Seven miRs, expressed in both adipose tissue and serum, were selected for validation phase in 34 women from a different cohort. An age/menopause-related increase of miRs-16-5p, -451a, -223-3p, -18a-5p, -19a-3p,-486-5p and -363-3p was found in the adipose tissue, but not in serum. MiR-19a-3p, involved in adipocyte development and estrogen signaling, resulted to be higher in HRT users in comparison with non-users. Among the identified targets, AKT1, BCL-2 and BRAF proteins showed lower expression in both HRT and No HRT users in comparison with premenopausal women. Unexpectedly, ESR1 protein expression was not modified although its mRNA was lower in No HRT users compared to premenopausal women and HRT users. Thus, both HRT and menopause appear to affect adipose tissue homeostasis via miR-mediated mechanism.

## INTRODUCTION

Human aging is a lifelong process characterized by a dynamic phenotype that changes over time, also associated with sex hormone differences in men and women [1]. During menopausal transition, concentration of circulating 17β-estradiol (E_2_) decreases concomitantly with an increase in follicle-stimulating hormone (FSH). Declining amounts of ovarian E_2_ lead to changes in the distribution of body fat [2] emphasizing the importance of ovarian E_2_ in regulating the lipid metabolism. The effects of E_2_ in adipose tissue are likely mediated through estrogen receptors among them ESR1 is known to affect whole-body metabolism as well as adipocyte cell growth and differentiation [3, 4]. Postmenopausal women adopting E_2_-based hormone replacement therapy (HRT) have healthier adipokine/cytokine profile and less centrally located body fat than women not using HRT [5, 6, 7]. This evidence is relevant since adipose tissue is metabolically active and, owing to its secretory activity, it is one of the main contributors to the crosstalk among different tissues [8]. Furthermore, the role of adipose tissue in human aging is central due to its influence on sustaining the pro-inflammatory microenvironment, the main premise underlying “inflammaging” and its propagation mechanisms [9, 10]. However, adipose tissue is not only responsive to fluctuations in the systemic levels of hormones, but it actively produces and secrets multitude of molecules including E_2_, other hormones, inflammatory factors and microRNAs (miRs).

MiRs are small RNAs that contribute to gene regulation by binding to their mRNA targets, a process that induces mRNA cleavage or seizure and, eventually, a possible reduction in the abundance of the functional target protein. Many of the miRs expressed by adipose tissue take part in adipogenesis and lipid homeostasis [11, 12, 13, 14]. Aging affects adipose tissue miR expression in humans [15], thus leading to alterations in miR target abundancy either locally or at systemic level eventually far from the site of exocytosis [16]. We have previously shown that specific circulating miRs [17, 18] and skeletal muscle miRs [19] of pre- and postmenopausal women were associated with serum E_2_ levels. These findings indicate that sex steroid hormones are part of miR-mediated mechanisms in aging women.

The aim of this study was to investigate the epigenetic changes, i.e. miR profiling expression, associated with age/menopause and HRT in two different specimens, i.e. blood serum and white adipose tissue, obtained from the same donors. Discovery phase was performed using miR card arrays on samples from premenopausal women (none used hormonal medication) and postmenopausal monozygotic (MZ) twin pairs discordant for HRT. Validation phase was performed in a larger different cohort of premenopausal women and of unrelated postmenopausal HRT and No HRT users. The most significant targets were identified both at mRNA and protein levels.

## RESULTS

### Participant characteristics

**Figure 1:**
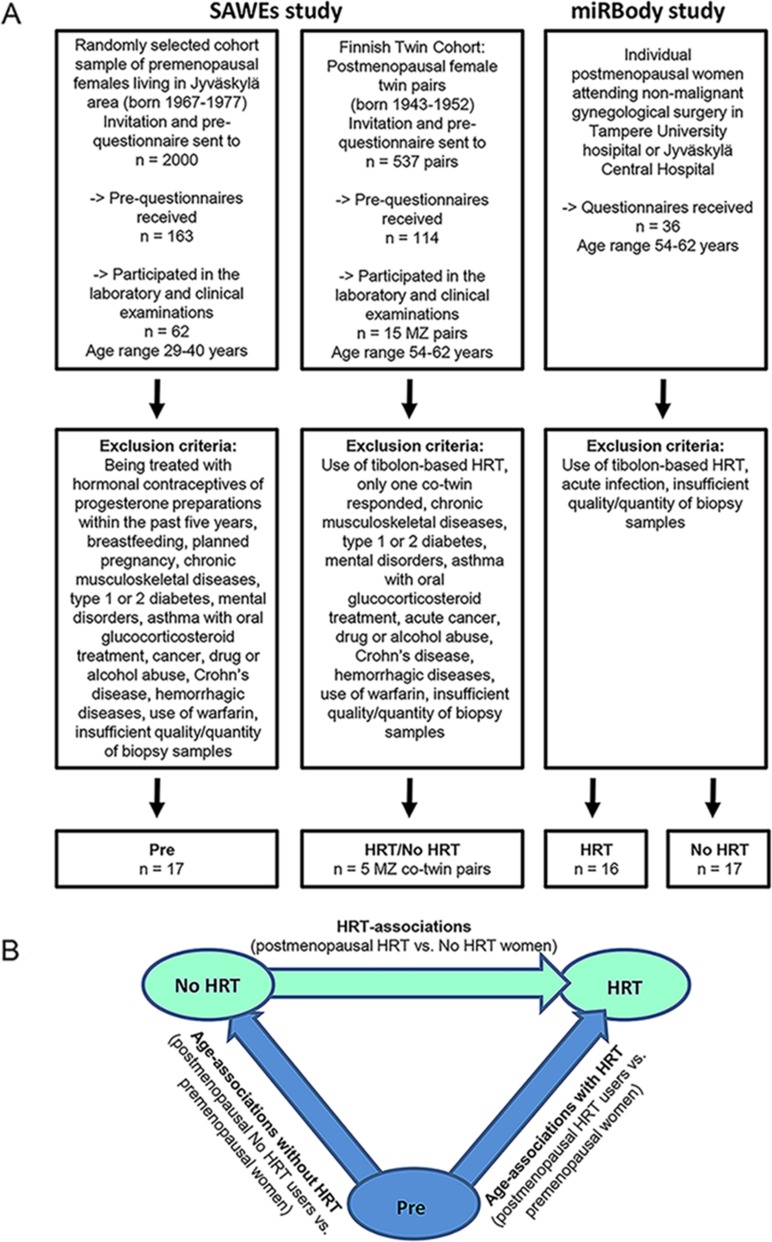
Recruitment of the participants and scheme of experimental design **Panel (A)** shows the flowchart of the recruitment process of the two different data sets used in the study. **Panel (B)** shows the investigated groups and comparisons. Pre = premenopausal women, No HRT = postmenopausal women without estrogen-based hormone replacement therapy, HRT = postmenopausal women with estrogen-based hormone replacement therapy. See also Supplementary Table 1.

Two different samples and data sets originating from 1) the SAWEs and 2) miRBody studies were analyzed (Figure 1A). The SAWEs study consists of a cross-sectional design with premenopausal women (Pre) without any E_2_-containing hormonal treatments, and a co-twin design of postmenopausal MZ twin sister pairs with approximately 7 years of E_2_-based HRT discordance (HRT and No HRT users), i.e. one sister was a current HRT user and the other had never used HRT. MiRBody is a cross-sectional study with a postmenopausal cohort of unrelated women who were age-matched with the SAWEs MZ twins and formed two groups, one using and the other not using HRT. The SAWEs samples were miR- profiled. The miRBody samples (postmenopausal HRT and No HRT women) and SAWEs samples (premenopausal women) were available for the validation of the profiling data. Figure 1B shows the scheme of comparisons among the identified three groups.

Table 1 shows anthropometric data, serum inflammatory markers and hormone levels measured in the different data sets among the groups and Supplementary Table 1 reports the clinical data. No differences in body composition measures or inflammatory markers were observed among the groups. Serum E_2_ and FSH levels differed, as expected, most significantly between the premenopausal women and No HRT users. In fact, E_2_ showed the highest values in the premenopausal women and the lowest values in the postmenopausal non-users and vice versa for FSH. Comparisons were also performed between the SAWEs and miRBody participants. The E_2_ levels of the postmenopausal No HRT group in the miRBody study were higher than those of the SAWEs No HRT women (p < 0.001) and the E_2_ levels of the SAWEs No HRT women differed significantly from the miRBody HRT women (p < 0.001). The differences between the No HRT groups might in part be due to the different methods used to analyze the E_2_ levels in these two studies (see methods). Otherwise, no group differences between postmenopausal SAWEs and miRBody women were detected.

**Table 1:** The participant characteristics

	Premenopausal women (SAWEs; n=17)	Postmenopausal HRT women (SAWEs; n=5)	Postmenopausal No HRT women (SAWEs; n=5)	Postmenopausal HRT women (miRBody; n=16)	Postmenopausal No HRT women (miRBody; n=17)
**Age (yrs)^#^**	32.9±3.3	57.4±1.5^*^	57.4±1.5^*^	57.7±2.9^*^	58.8±3.0^*^
**Years on HRT**	-	8.4±4.7	-	6.6±6.3	-
**BMI (kg/m2)**	27.1±5.7	26.6±3.3	27.3±5.6	27.7±4.6	27.9±3.8
**Fat %**	32.3±9.2	31.6±7.3	33.3±9.3		
**LBM (kg)**	45.5±4.6	45.1±2.0	44.2±4.0		
**CRP (mg/l)**	1.4±1.7	1.3±1.1	1.3±0.7	2.4±2.2 (n=15)	3.5±3.8 (n=15)
**HGB (g/l)**	136.5±7.1	146.0±8.1^*$^	138.8±12.4	138.7±10.1 (n=12)	135.0±10.1 (n=11)
**WBC (e9/l)**	5.7±1.3	5.9±1.1	5.7±1.8	6.4±1.5 (n=12)	6.1±1.8 (n=11)
**E_2_ (pmol/l)^#^**	355.4±288.5	250.4±296.4^*†^	23.8±10.6^*^	284.4±177.4^$†^	118.4±46.9^*†^
**FSH (IU/l)^#^**	6.0±2.3	39.8±32.0^*^	75.1±46.5^*^	32.9±24.6^*^	58.2±28.4^*^

See also Supplementary Table 1.

Results are shown as mean ± S.D. For parametric variables, independent samples *T*-test was used to compare the postmenopausal groups with the premenopausal group and to compare mirBody postmenopausal HRT group with the No HRT group, while paired samples *T*-test was used to compare the SAWEs HRT women with the No HRT co-twins. For non-parametric variables, ^#^Mann Whitney *U* test was used to compare the postmenopausal groups with the premenopausal group and to compare mirBody postmenopausal HRT group with the No HRT group, while Wilcoxon matched pair signed-rank test was used to compare the SAWEs HRT women with the No HRT co-twins. Significant comparisons between the studied groups: ^*^P<0.05 compared to the Premenopausal group; ^†^P<0.05 compared to the SAWEs Postmenopausal No HRT group; ^$^P<0.05 compared to the miRBody No HRT group. BMI: body mass index, LBM: lean body mass (fat free), CRP: high sensitivity C-reactive protein, HGB: hemoglobin, WBC: white blood cell count, E_2_: 17β-estradiol, FSH: follicle-stimulating hormone.

### miR profiling and discovery phase

**Figure 2:**
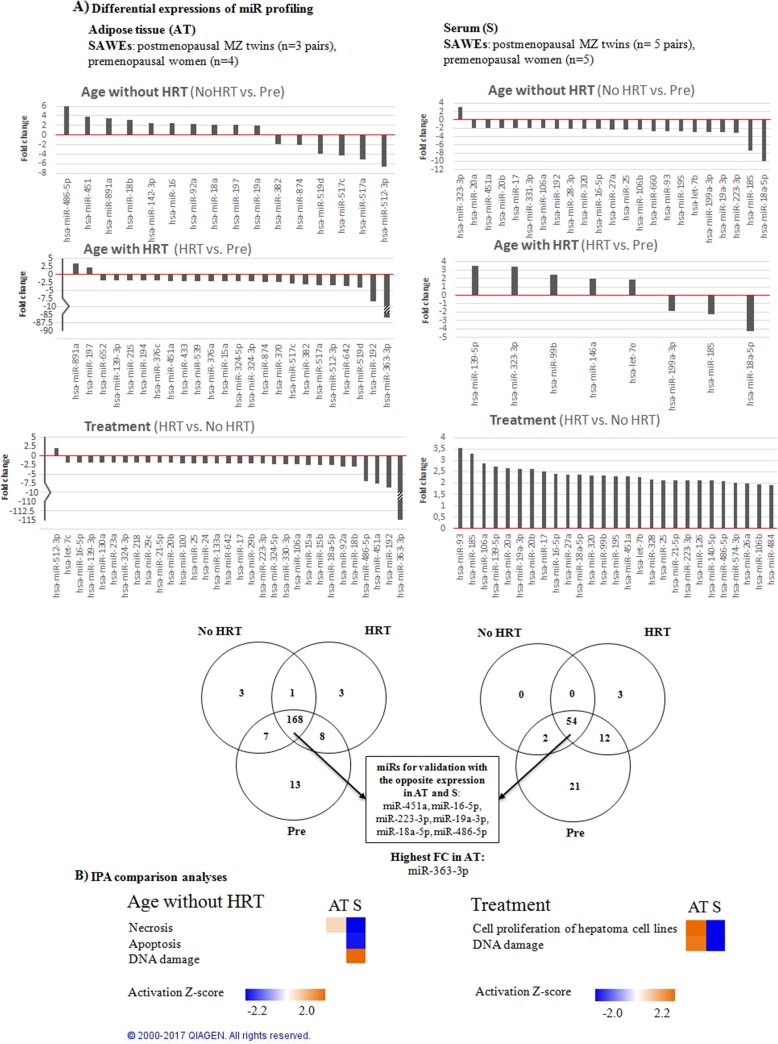
MiR profiling of adipose tissue and serum-derived miRs **(A)** Discovery phase of the miRs performed on arrays for both tissues (FC ≥ ±1.9). Numerical FC values for the comparisons are specified as supplementary data (Supplementary Table 2). All the comparisons, i.e. No HRT *vs* Pre, HRT *vs* Pre, HRT *vs* No HRT, were taken into account in both 10 adipose tissue (on left) and 13 blood serum samples (on the right). Venn diagram presents the numbers of all the detected miRs and those chosen for RT-qPCR validation. **(B)** IPA comparison analyses were performed between adipose tissue and serum miRs in each comparison (FC ≥ ±1.9, Z-activation score ≥ ± 2.0). Results show the differences between the two tissues in terms of diseases and biological functions. Orange indicates up- and blue downregulation.

TaqMan human card array containing 377 miRs for profiling in 13 women (including twin pairs) was used. Ten subcutaneous adipose tissue and 13 serum samples obtained from the premenopausal women and postmenopausal MZ twin sister pairs of the SAWEs study were analyzed. In total, 203 miRs in the adipose tissue and 92 miRs in the serum (Figure 2A) were detected. Three types of comparisons were performed as follows: 1) Postmenopausal No HRT group *vs*. premenopausal women in order to identify hormonal aging-associated differences. 2) Postmenopausal HRT group *vs*. premenopausal women in order to identify hormonal aging-associated differences along with E_2_-supplementation. 3) Postmenopausal HRT group *vs*. postmenopausal No HRT group in order to identify HRT-associated differences at the same genetic background. MiRs with fold change (FC) values ≥ 1.9 are shown in Figure 2A. A list of all the FCs obtained is specified as supplementary data (Supplementary Table 2). The experimental design (Figure 1B) and miR filtering procedure, as detailed in Figure 2A, yielded the following results: Age associations without HRT (No HRT *vs*. Pre) were defined for 16 and 23 miRs in adipose tissue and serum, respectively. Age associations with HRT (HRT *vs*. Pre) were defined for 24 and 8 miRs in adipose tissue and serum, respectively. HRT associations (HRT *vs*. No HRT) were defined for 31 and 27 miRs in adipose tissue and serum, respectively.

Ingenuity Pathway Analysis (IPA) was performed to identify the potential biological processes and possible interactions with the profiled adipose tissue and serum-derived miRs taking into account all the comparisons among the groups (Figure 2B). The analyses predicted differences in up- or downregulated biological functions between adipose tissue and serum. In No HRT *vs*. Pre -comparison biological processes, as necrosis, apoptosis and DNA damage were predicted to be affected. In HRT *vs*. No HRT comparison, the predicted pathways were cell proliferation of hepatoma cell lines and DNA damage. HRT *vs.* Pre -comparison showed no statistically significant predictions in any biological processes. The identified biological processes revealed opposite patterns in the adipose tissue and serum samples.

### Validation of the miR profiling

**Figure 3:**
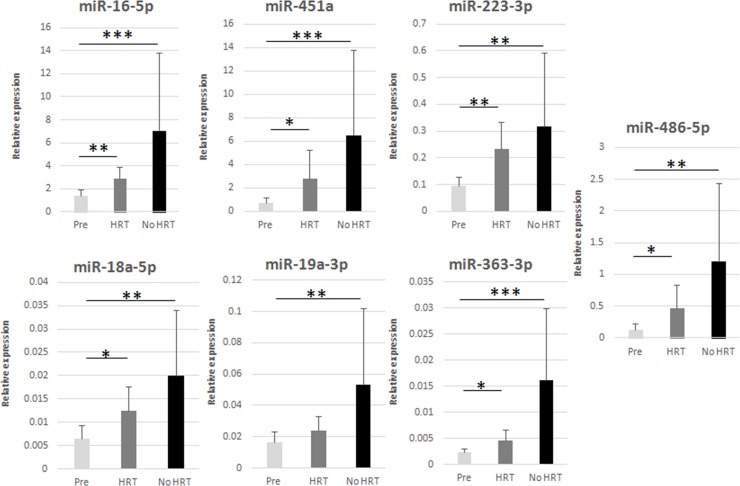
RT-qPCR validation of selected miRs in adipose tissue ^*^ p < 0.05, ^**^ p < 0.01, ^***^ p < 0.001. Pre = premenopausal women (n=12), HRT = postmenopausal HRT users (n=9), No HRT = postmenopausal non-users (n=12). Results are shown as mean ± SD. Data were normalized by RNU44 expression levels.

**Figure 4:**
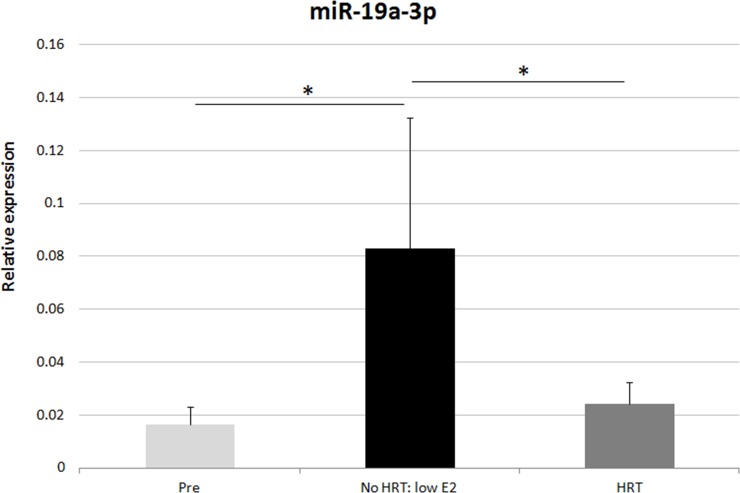
RT-qPCR validation of miR-19a-3p in adipose tissue Relative expression of miR-19a-3p in all the groups considering No HRT women with low E_2_ level (53.6-99.1 pmol/l) ^*^ p < 0.05, Pre = premenopausal women (n=12), HRT = postmenopausal HRT users (n=9), No HRT = postmenopausal non-users (n= 6). Results are shown as mean ± SD. Data were normalized by RNU44 expression levels.

MiR profiling was validated in 34 women by means of RT-qPCR. Sixty-seven samples (33 adipose tissues, 34 serum) from the premenopausal women (SAWEs) and the independent clinical cohort of postmenopausal HRT and No HRT users (miRBody) were analyzed. We selected those miRs detected in all measured samples. Further, we selected 6 statistically relevant miRs (FC ≥1.9) expressed in both adipose tissue and serum, obtained from the same donor. Interestingly, these selected miRs (miR-16-5p, miR-451a, miR-223-3p, miR-18a-5p, miR-19a-3p, miR-486-5p) were by chance expressed in the opposite directions considering adipose tissue and serum for each group comparison. One additional miR, showing the highest FC in adipose tissue (miR-363-3p), was also selected, as reported in Figure 2A. In the adipose tissue (Figure 3) the main results were the following: miRs-16-5p, -451a, -223-3p, -18a-5p, -19a-3p, -363-3p, and miR-486-5p were more expressed with age/menopause (No HRT *vs*. Pre: p < 0.001; p < 0.001; p = 0.003, p = 0.003; p = 0.006; p < 0.001; p = 0.002, respectively). These results were mainly in accordance with miR profiling (except miR-223-3p and miR-363-3p). The same miRs were confirmed to be more expressed with age and HRT use (HRT *vs*. Pre: p = 0.002; p = 0.014; p = 0.009; p = 0.024; p = 0.036; p = 0.039, respectively), except miR-19a-3p. These results were not in accordance with miR profiling excluding the case of miR-16-5p. MiR-19a-3p resulted to be sensitive to HRT when No HRT women, having E_2_ levels lower than 110 pmol/l were considered, thus excluding No HRT with highest E_2_ values. E_2_ concentration of 110 pmol/l represented the lowest E_2_ value found in the HRT group. It was essential to avoid overlapping between HRT and No HRT groups in terms of E_2_ serum values to find differences between the two groups. In fact, the analysis revealed significantly higher miR-19a-3p levels in the postmenopausal low-E_2_ No HRT than HRT groups (p = 0.043, Figure 4).

In serum samples of the same women, single miR analyses of miRs-16-5p, -451a, -223-3p, -18a-5p, -19a-3p, -363-3p, and miR-486-5p by RT-qPCR did not confirm data obtained by the miR profiling. No significant differences were observed. Therefore, further analyses of the miR targets were performed only in adipose tissue.

### Adipose tissue miRs associate with age and circulating hormones

**Figure 5:**
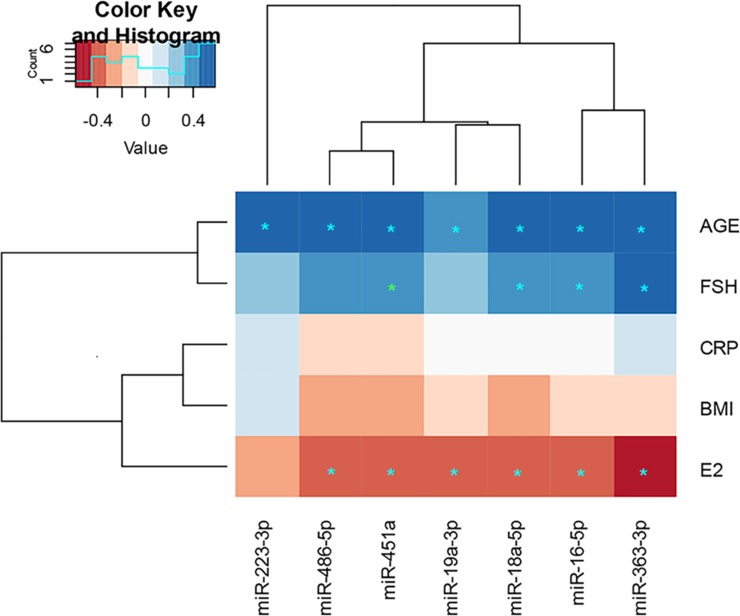
Clustered heatmap including the validated adipose tissue miRs and their associations with serum hormones, CRP and BMI ^*^ p < 0.05, ^*^ p < 0.05 with FDR correction. The numerical values of the Spearman correlation, P-values and FDR corrected P-values are specified as supplementary data (Supplementary Table 2). FSH = follicle-stimulating hormone, CRP = high sensitivity C-reactive protein, BMI = body mass index, E_2_: 17β-estradiol. See also Supplementary Table 3.

A clustered correlation heatmap was created to determine the associations of the validated adipose tissue miRs with age, body mass index (BMI) and circulating high sensitivity C-reactive protein (CRP), E_2_ and FSH levels (Figure 5). All the adipose tissue miRs correlated positively with age (false discovery rate corrected p-value -FDR < 0.05) and all, except miR-223-3p, negatively with serum E_2_ levels (FDR < 0.05). In addition, miR-18a-5p, -16-5p and -363-3p showed positive correlations with serum FSH levels. None of these miRs were significantly associated with BMI or CRP. Numerical details of the correlations are specified as supporting information (Supplementary Table 3).

### mRNA and protein expressions of miR targets in adipose tissue

**Figure 6:**
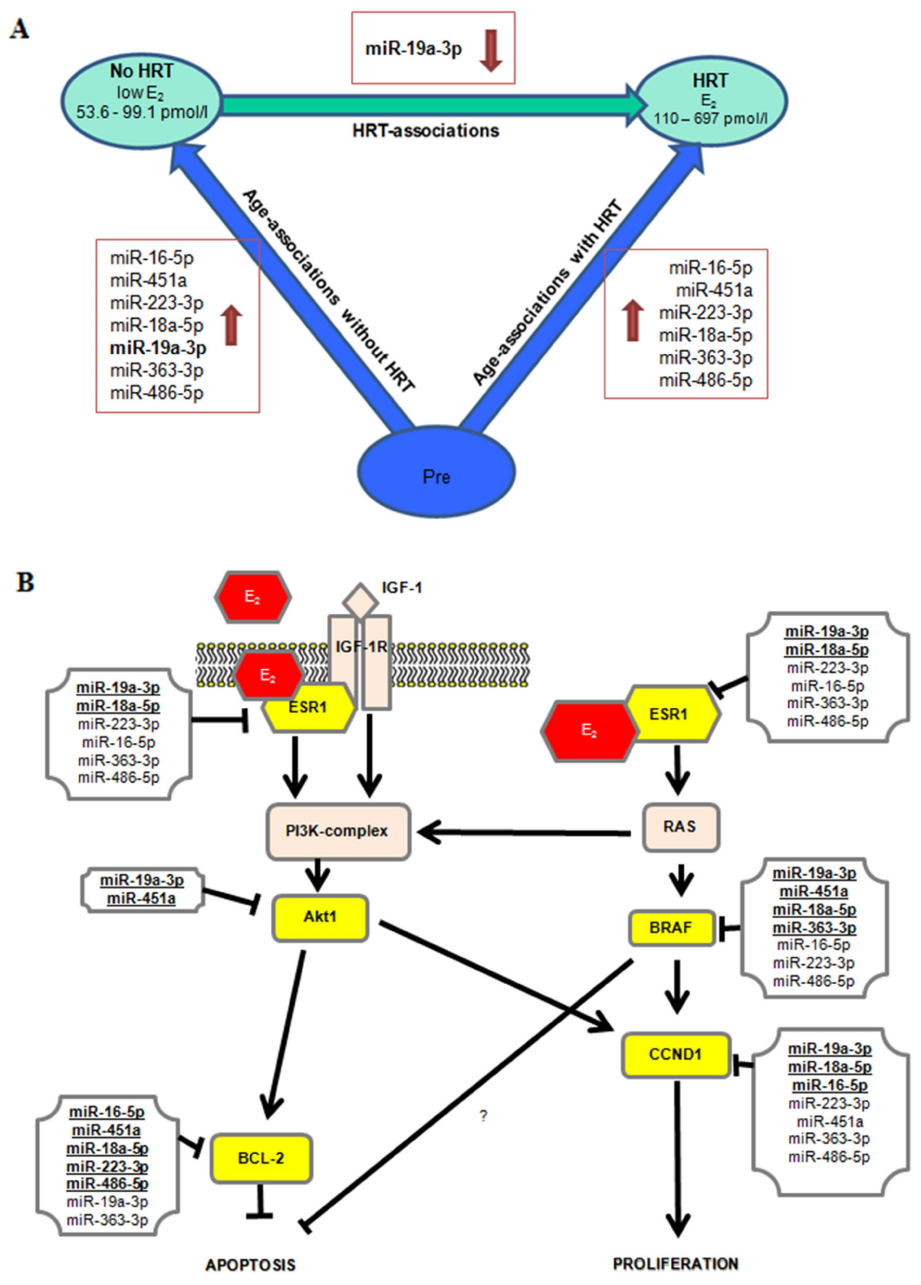
Overall miRs results and their targets involved in estrogen signaling pathway **(A)** Overall miRs results considering all the comparisons and No HRT women with lower E_2_ level. **(B)** Experimentally validated miR-target pairs are bolded and underlined. The other miRs indicate putative miR-target pairs. Currently selected targets are highlighted in yellow. Data is obtained from http://diana.imis.athena-innovation.gr/DianaTools/index.php and http://zmf.umm.uni-heidelberg.de/apps/zmf/mirwalk2/. References for experimentally validated miR-target pairs are published [24, 25, 26, 27, 48, 49, 50, 51, 52, 53, 54, 55].

**Figure 7:**
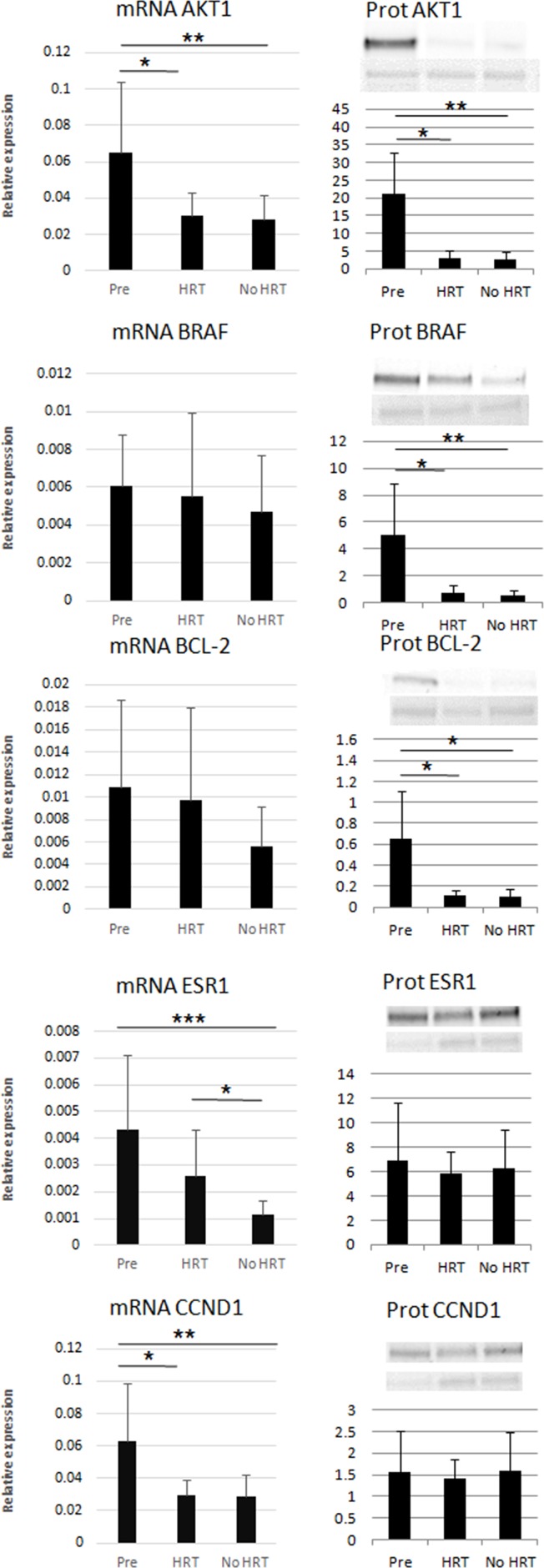
Relative mRNA expression and protein levels of the miR targets ^*^ p < 0.05, ^**^ p < 0.01, ^***^ p < 0.001. Pre = premenopausal women (n_mRNA_=12, n_prot_=5), HRT = postmenopausal HRT users (n_mRNA_=9, n_prot_=5), No HRT = postmenopausal non-users (n_mRNA_=12, n_prot_=5). Results are shown as mean ± SD. In the left column mRNAs and in the right column protein data are reported. mRNA data were normalized against GADPH expression levels. In the right column, the upper western blots show the investigated proteins and the lower blots show the Ponceau dye (60 kDa) used for normalization procedure.

A bioinformatic approach, based on experimentally validated and published miR-target pairs, was applied for the currently validated miRs (Figure 6A). Thus, the common targets considering at least two miRs targeting the same mRNA including ESR1, AKT1, BRAF, BCL-2 and CCND1 were identified (Figure 6B). Both mRNA and protein levels were measured in adipose tissue samples for all the identified targets (33 mRNA and 15 protein samples), as described in Figure 7. ESR1, AKT1 and CCND1 mRNAs were less expressed with aging/menopause (No HRT *vs*. Pre; p < 0.001, p = 0.005, p = 0.003, respectively). At protein level, AKT1, BRAF and BCL-2 were significantly less abundant in postmenopausal women (p = 0.005, p = 0.003, p = 0.028, respectively). AKT1 and CCND1 mRNAs were also less expressed with aging/HRT use (HRT *vs*. Pre; p = 0.023, p = 0.014, respectively). At protein level, AKT1, BRAF and BCL-2 were significantly less abundant in HRT in comparison with premenopausal women (p = 0.013, p = 0.020, p = 0.024, respectively). ESR1 mRNA was found to be more expressed with HRT treatment (HRT *vs*. No HRT; p = 0.026) whereas at protein level ESR1 values were similar in all groups.

## DISCUSSION

Aim of the study was to focus on epigenetic changes associated with a crucial event of woman's reproductive life, i.e. menopause, when evolution force is predicted to decline, accordingly with some theories of aging [20] and likely, when aging trajectories move towards either the onset of age-related diseases or healthy status preservation [21]. To better disentangle the role of estrogen, a group of postmenopausal women using E_2_-based HRT was studied along with healthy pre- and postmenopausal women without any E_2_-based medications. MiR expression was selected to be a relevant marker for tissue aging [22], being also a type of “epigenetic communication” among organs and tissues, when blood circulation is comprised. In fact, both adipose tissue and serum from the same individual were analyzed. Initially, we adopted an exploratory miR profiling approach to determine the differences of miR expression, assuming causality due to aging or menopausal status and/or postmenopausal HRT treatment. We detected 203 miRs in the adipose tissue and 92 in the serum samples. Functional analysis with IPA predicted necrosis, apoptosis and DNA damage-related biological processes to be the main downstream mechanisms affected by these miRs with menopause status. The IPA results support the general hypothesis that inflammaging is fueled by the age-related production of cell debris or misplaced cell-molecules released during repair or damage processes, along with circulating miRs [23]. Previous data obtained on monozygotic twin pairs discordant for HRT showed a correlation between blood circulating inflamma-miRs and a pro-inflammatory cytokine in No HRT but not in HRT group, suggesting possible anti-aging effects of HRT [17].

We selected for validation 6 miRs found to be present in both the adipose and serum samples with opposite trends of expression (considering all the comparisons among the three groups of women: miR-16-5p, miR-451a, miR-223-3p, miR-18a-5p, miR-19a-3p, miR-486-5p) and the most highly expressed miR in adipose tissue (miR-363-3p). In line with the results obtained by the profiling, RT-qPCR validation confirmed most of the age-menopause associations of miRs increase in adipose tissue. We demonstrated that all the selected miRs increase with age or menopausal status and the same miRs, except miR-19a-3p, were also found highly expressed in HRT users, thus suggesting no effect of HRT on these selected miRs. Validated miRs were shown to target ESR1, AKT1, BCL-2, BRAF and CCND1, thereby indicating their role in the regulation of adipocyte cell fate, death or proliferation.

Despite the profiling results, the selected miRs were not differentially expressed in the serum, suggesting not only a specific effect of HRT treatment on adipose tissue, but also no relations of age and HRT with the selected miRs in the circulation. Validation phase is crucial, since card array technology sometimes can produce bias especially when studying circulating miRs. Recently, we demonstrated that age/menopause and HRT differentially modulate the expression of specific miRs shuttled in circulating exosomes [18], by means of next generation sequencing (NGS). To this regard, the high resolution of NGS confirms the absence of changes in the currently selected miRs, thus reinforcing the result that hormonal aging and HRT have tissue-specific effects that do not “propagate” in the blood, and do not affect the level of the circulating miRs, at least for those selected.

Importantly, we have shown that miR-19a-3p is sensitive to HRT. In fact, HRT women have a level of miR-19a-3p similar to younger premenopausal controls, while No HRT users, with lower E_2_ blood levels, have a higher expression of miR-19a-3p, suggesting an anti-aging effect of HRT in terms of regulation of this miR in the adipose tissue.

Of note, miR-19a-3p and -18a-5p have previously been shown to target ESR1 [24, 25, 26, 27]. These miRs are part of a miR-17-92 cluster, which has been shown to participate in the regulation of adipocyte development by accelerating preadipocyte differentiation [28]. Our results showed the opposite expression patterns between miR-18a-5p; -19a-3p and ESR1 mRNA target, as expected. MiR-17-92 cluster was previously demonstrated to be involved in aging process and a decrease of miR-19b was shown in various *in vitro* or *ex vivo* cell models [29, 30]. We observed a trend of miR-19a-3p increase with age/menopause along with lower serum E_2_ concentration. These apparently opposite trends are likely due both to the different type of analyzed tissue/cell and the different miR-17-19 cluster members (miR19a *vs* miR-19b), which may have different targets and functions.

Furthermore, our results indicate that miR-223-3p, -16-5p, -363-3p and -486-5p, which are predicted to target ESR1 based on their complementary sequence structure, also may contribute to the regulation of ESR1 expression, owing to their similar expression pattern with miR-18a-5p and -19a-3p and negative correlation with E_2_ (except for miR-223-3p) as shown in the hierarchical clustering analysis. As expected, the levels of the miRs targeting ESR1 have an opposite trend with the amount of ESR1 mRNAs in the adipose tissue. Nevertheless, ESR1 protein levels appear well preserved in all groups, thus suggesting other regulatory layers [31] and the great capacity of adipose tissue to maintain the expression of a key regulatory receptor. In fact, the expression of ESR1 is found different depending on the type and site of adipose tissue in the same individual [32] under the same level of blood estrogen. These data mean that a tight ESR1 protein regulation occurs and we confirm that ESR1 protein is also preserved with aging/menopause. Further, the possible interaction of various species of small or long non-coding RNAs or circular RNAs were not investigated here [33, 34]. Indeed, the low number of protein samples and the potential effect of local production of E_2_ in adipose tissue, which we had no opportunity to investigate here, should be acknowledged as possible limitations of this work.

As far as the other validated miRs are concerned, miR-363-3p and miR-486-5p have previously been shown to be involved in adipose tissue remodeling. A study performed by Chen and colleagues [11] demonstrated that miR-363 is downregulated during adipogenic differentiation in the adipose tissue-derived stromal cells. Based on those findings, our data support that adipose tissue status from post-menopausal women, having the highest miR-363-3p expression, is likely related with reduced differentiation capacity. This finding is reinforced by the common notion that a younger tissue is more prone to proliferate and differentiate than older tissue. Further, the expression of miR-486-5p in human adipose tissue-derived mesenchymal stem cells has been shown to induce replicative senescence [35]. Our study reveals that higher age is related with higher expression of miR-486-5p in adipose tissue, thereby emphasizing the possible role of the menopause on the cellular homeostasis or early phase of replicative senescence in the tissue.

To our knowledge, no previous studies has been focused on miR-451a expression in adipose tissue. Instead, the association of miR-451a with aging has been identified in the skeletal muscle of monkeys [36], where miR-451a levels were shown to be higher with higher age. In addition, decreased expression of miR-451a in non-alcoholic steatohepatitis human liver was associated with an increase in pro-inflammatory cytokines [37], suggesting its role in inflammation. In the current study, miR-451a was highly expressed with aging in adipose tissue independently of HRT use. Interestingly, a high age-related miR level appears to be a common trend shared by other metabolic organs such as the human liver, as recently reported [22], suggesting a causal association between the hormonal age-decline and the general increase of miRs.

MiR-16-5p and miR-223-3p are widely studied in different cellular models. Age-related changes of miR-16-5p have been shown to be associated with vascular and neurodegenerative diseases and B-cell function [38, 39] whereas, with advanced age, an increase of miR-223-3p in inflammatory cells has been reported [40]. MiR-223-3p was also found to regulate macrophage activation resulting in the suppression of pro-inflammatory responses in the adipose tissue of mice [41]. In the current study, the highest expression of both miRs was found in the adipose tissue of postmenopausal women without HRT, strengthening their association with aging. However, the possible contribution to the level of miR-223-3p of adipose tissue macrophages cannot be excluded. In addition, we have previously demonstrated an association between HRT and miR-223-3p in the skeletal muscle of these same postmenopausal HRT-discordant MZ twins, thus suggesting tissue-specific regulation of miR-233-3p expression [19].

ESR1 signaling pathway in adipose tissue is likely to be followed by the activation of AKT1, leading in turn to more proliferative or anti-apoptotic processes. AKT1 has also been previously linked to estrogen signaling [42]. Our results showed that postmenopausal women have lower amounts of AKT1 in their subcutaneous adipose tissue than premenopausal women. This age-association was consistent for both the mRNA and protein levels independently of HRT use. AKT1 affects cellular homeostasis and it is considered as a survival factor suppressing apoptosis. The role of AKT1 in human adipocytes was previously demonstrated [43] and authors showed that AKT1 takes part in insulin-induced metabolic signaling. In the current study, the lower amount of AKT1 in the postmenopausal women could indicate a weakened sensitivity to insulin and reduced proliferative status. Further, E_2_ is able to induce BCL-2 and CCND1 in breast cancer cells [44] and they are downstream from AKT1. We showed that BCL-2, an anti-apoptotic protein, is less abundant with aging suggesting that the rate of apoptosis could be accelerated in older women. On the contrary, CCND1 mRNA levels were negatively associated with age. However, the proteins did not differ among the groups, suggesting a more complex post-transcriptional regulation. The mRNA expression of BRAF, a molecule regulating cell growth independently of AKT1, did not differ among the groups. However, its protein was lower with higher age, suggesting, also in this case, further levels of regulation. These findings indicate lower proliferation capacity of adipose tissue in postmenopausal than premenopausal women being in line with previous studies showing sex-dependent differences in healthy adipose tissue remodeling [45]. Further, our results also support lower expression of anti-apoptotic mediators with postmenopausal age.

Altogether, our data indicate that menopausal transition acts as a crossway between apoptotic and proliferative signaling. Lower levels of specific proteins, i.e. AKT1, BCL2 and BRAF, after menopause suggest that the proliferative activity is likely slowed down in the subcutaneous adipose tissue obtained from the abdominal region. Based on the current findings, we can hypothesize that adipocytes are addressed towards deficiency in cellular remodeling and/or early phase of cellular senescence following menopause. The subcutaneous adipose tissue, even if not representative of all the different types of fat in humans, shows an age-related increase of miR expressions involved in the estrogen-signaling pathway.

The interplay of age, systemic E_2_ levels and tissue-specific miRs highlights the critical role of HRT that partially slows down the effects of menopausal estrogen decline in a tissue-related manner and specifically on miR-19a-3p. HRT is currently used by over 100 million women worldwide thus its application as an anti-aging therapy to counteract the development of osteoporosis, cardiovascular diseases and metabolic disorders and likely increasing the healthy life span is definitively relevant. However, longitudinal studies with greater numbers of participants need to confirm the proposed causality of the present findings and possibly, to evaluate the miRs not included in the validation phase.

## MATERIALS AND METHODS

### Experimental design

The current study is based on two different sample and data sets as presented in Figure 1A, 1B: The former is named: Sarcopenia and Skeletal Muscle Adaptation to Postmenopausal Hypogonadism: Effects of Physical Activity and Hormone Replacement Therapy in Older Women – a Genetic and Molecular Biology Study on Physical Activity and Estrogen- related Pathways (SAWEs-study). The latter is named: Circulating microRNAs and body composition (miRBody-study). The recruitment process and exclusion criteria for participation for both studies are also presented in the figure.

Briefly, the SAWEs-study investigated a group of healthy premenopausal women (n=17, 32.9 ± 3.3 years) with a natural menstrual cycle and no estrogen/progesterone-based treatments for at least the previous 5 years [46] and a group of postmenopausal MZ twin sister pairs (n=5 pairs, 57.4 ± 1.5 years) discordant for E_2_-based HRT (mean duration of HRT use 6.9±4.1 years) [6]. The miRBody-study investigated 33 independent postmenopausal women either using estrogen-based HRT (mean duration of HRT use 6.6±6.3 years, n=16, age 57.7±2.9 years) or not (n=17, age 58.8±3.0 years). The study protocols were approved by the Ethics Committee of the Central Finland Health Care District (SAWEs: 7.6.2006 and 22.11.2006 E0606/06; miRBody: 3.2.2015 1U/2015). All the study participants gave their written informed consent. The study was conducted according to the guidelines of the Declaration of Helsinki.

### Participant characteristics

BMI was calculated from body weight and height (kg/cm^2^). Body fat percentage and lean body mass (LBM) were measured with bioelectrical impedance (inBody 720, Biospace Co. Ltd., Seoul, Korea). Serum E_2_ levels were measured using an extraction radioimmunoassay (SAWEs) [6] and with solid-phase, chemiluminescent immunometric assay (miRBody) which was also used for the serum CRP and FSH measurements (Immulite 1000, Diagnostic Products, Los Angeles, CA, USA).

### Sampling

In the SAWEs study, adipose tissue biopsies were obtained from below the navel by a physician with the needle-aspiration method. Blood traces were cleaned with 0.9 % NaCl and samples were snap frozen in liquid nitrogen and stored at -80°C until further analysis. The samples used in the mirBody-study were obtained from abdominal subcutaneous adipose tissue in the proximity of the navel during gynecological surgery. Samples were stored in All Protect Reagent (Qiagen) at -20°C until further analysis. All SAWEs blood samples were taken between 7.00 to 9.00 am after overnight fast. Furthermore, the samples from the premenopausal women were collected during the first five days of the menstrual cycle to obtain the lowest E_2_ concentrations. Unfortunately, due to the varying times of the surgeries, the blood samples taken from the miRBody women were not taken under overnight fast but instead followed the routine standardized practices of the operating hospital. All blood samples, were allowed to clot for 30 min at room temperature before serum separation by centrifugation at 4000 rpm. Serum samples were stored at -80°C in 0.5 ml aliquots until further analysis.

### RNA extraction

Total RNA (~20 mg of tissue) was extracted from adipose biopsies with a mirVana miRNA isolation kit (Ambion, by Life Technologies, NY, USA) according to manufacturer's protocol. One hundred μl of serum sample was used for the extraction of total RNA with Total RNA Purification kit (Norgen Biotek Corp, Thorold, Canada). The hemolysis has been controlled through visual inspection also comparing a control sample (without hemolysis evaluated by the ratio of miR-23a and miR-451) [47]. In addition, 20 fmol of spike-in cel-miR-39 (Qiagen, Hilden, Germany) was added to the serum samples at the lysis step to control for the RNA extraction efficiency. Recovery of the spike-in cel-miR-39 was constant with a mean Ct value of 16.6 ± 0.6.

### MicroRNA profiling

To assess global miR expression, 10 SAWEs adipose tissue samples (4 premenopausal, 3 HRT users, 3 non-users) and 13 serum samples (4 premenopausal, 5 HRT users, 4 non-users) were screened using a TaqMan human MicroRNA Array A (Applied Biosystems, by Life Technologies, NY, USA) containing 377 of the most common human miR assays. MiR profiling protocol is described in details in Supplementary Materials.

### Confirming the profiling results

For RT-qPCR validation, six miRs (miR-16-5p, miR-451a, miR-223-3p, miR-18a-5p, miR-19a-3p and miR-486-5p) expressed in both adipose tissue and serum samples across all the participants were selected from the profiling analyses, being not only expressed in both tissues, but having also opposite pattern of expression. In addition, miR-363 was also validated owing to its extreme FC in adipose tissue comparing all the groups. Samples from the premenopausal SAWEs women (n=9) and postmenopausal miRBody women (HRT: n=9, No HRT: n=12) were used for validation (see in details in Supplementary Materials).

### MiR target analyses from adipose tissue

Based on the literature, previously validated miR targets were analyzed. SAWEs samples from the premenopausal women (n=12) and mirBody samples from the postmenopausal women (HRT; n=9, No HRT; n=12) were used to assess the mRNA target expressions by RT-qPCR. The protein abundancy was analyzed by Western blotting (n=5/each group). RNA and protein analyses are described in details in Supplementary Materials.

### Statistical analysis

The normal distribution of the studied variables, was tested by the Shapiro-Wilk test and the group comparisons for anthropometric, serum inflammatory markers and hormones were performed either with Independent samples *T* test and paired samples *T* test (within twin pairs) for parametric variables, or with Mann Whitney *U* test and Wilcoxon signed-rank test (within twin pairs) for non-parametric variables. The Kruskall-Wallis test for non-parametric variables was used for comparisons of miR expressions across groups. MiR profiling data with FC values greater than 1.9 were used in the IPA comparison analyses (Qiagen) and an activation Z-score greater than 2 was considered statistically significant.

Venn diagram were obtained with http://bioinfogp.cnb.csic.es/tools/venny/index.html. Diana tools (http://diana.imis.athena-innovation.gr/DianaTools/index.php) and miRWalk (http://zmf.umm.uni-heidelberg.de/apps/zmf/mirwalk2/) were used for identifying the common validated target genes of the analyzed miRs. Spearman's rank correlation coefficient was used for the correlation analyses and R packages “gplots” and “RColor-Brewer” were used to create the clustered heatmap. P-values less than 0.05 were considered statistically significant.

## SUPPLEMENTARY MATERIALS FIGURES AND TABLES





## Abbreviations

ESR1: estrogen receptor 1
AKT1: AKT serine/threonine kinase 1
BRAF: B-Raf proto-oncogene, serine/threonine kinase
BCL-2: B-cell lymphoma 2
CCND1: cyclin D1
