# Supplementary material for: Menopause and adipose tissue: miR-19a-3p is sensitive to hormonal replacement
Source: Oncotarget. 2017 Dec 18;9(2):2279–94. doi: 10.18632/oncotarget.23406 (PMC5788639; doi:10.18632/oncotarget.23406)
Supplement: Supplementary file 1 [file oncotarget-09-2279-s001.pdf]

Menopause and adipose tissue: miR-19a-3p is sensitive to hormonal replacement

SUPPLEMENTARY MATERIALS

Experimental design

The current study is based on two different sample and data sets (Figure 1): 1. Sarcopenia and Skeletal Muscle Adaptation to Postmenopausal Hypogonadism: Effects of Physical Activity and Hormone Replacement Therapy in Older Women – a Genetic and Molecular Biology Study on Physical Activity and Estrogen- related Pathways (SAWEs-study); 2. Circulating microRNAs and body composition (miRBody-study). The recruitment process and exclusion criteria for participation for both studies are presented in Figure 1A and the analysis strategy of the current miR-study in Figure 1B. Briefly, the SAWEs-study investigated a group of healthy premenopausal women in a cross-sectional study design and a group of postmenopausal MZ twin sister pairs in a co-twin discordance design. The premenopausal women (n=17, 32.9±3.3 years) used in the study comprised healthy women, with a natural menstrual cycle and no estrogen/progesterone-based treatments for at least the previous 5 years. The premenopausal women's group was used in the miR profiling, miR validation and target mRNA/protein analyses. The premenopausal women have previously been described in details [1]. The group of ten postmenopausal SAWEs women comprise of healthy MZ co-twin sister pairs (n=5 pairs, 57.4±1.5 years), discordant for E_2-based HRT (mean duration of HRT use 6.9±4.1 years). The HRT users either used only E_2-based (1-2 mg) treatment or combined estrogenic (1-2 mg) + progestogenic treatment. These women were originally recruited from the Finnish Twin Cohort [2] and included the twin pairs born in 1943-1952 (n=537 pairs). To be eligible for the SAWEs-study, both co-twins had, among other inclusion criteria, to be willing to participate and donate adipose tissue biopsy samples from the abdominal region. The twin study design has previously been described in details [3].

The miRBody-study investigated 33 independent postmenopausal women either using estrogen-based HRT (mean duration of HRT use 6.6±6.3 years, n=16, age 57.7±2.9 years) or not (n=17, age 58.8±3.0 years). Adipose tissue and serum samples were collected from the abdominal region from women having no acute infections while attending non-malignant gynecological surgery either of two Finnish hospitals: the University Hospital of Tampere or the Central Finland Central Hospital in Jyväskylä (2015-2016). Adipose tissue samples taken from these women were used to confirm the miR profiling results and investigate potential miR targets (mRNA and proteins).

After being informed of the possible risks involved in the physiological measurements and biopsy sampling, all the study participants gave their written informed consent, including permission for the use of the gathered data for research purposes only. The study protocols were approved by the Ethics Committee of the Central Finland Health Care District (SAWEs: 7.6.2006 and 22.11.2006 E0606/06; miRBody: 3.2.2015 1U/2015). The study was conducted according to the guidelines of the Declaration of Helsinki.

MicroRNA profiling

To assess global miR expression, 10 SAWEs adipose tissue samples (4 premenopausal, 3 HRT users, 3 non-users) and 13 serum samples (4 premenopausal, 5 HRT users, 4 non-users) were screened using a TaqMan human MicroRNA Array A (Applied Biosystems, by Life Technologies, NY, USA) containing 377 of the most common human miR assays. RNA was converted to cDNA by priming with mixture of looped primers (MegaPlex kit, Applied Biosystems by Life Technologies) according to manufacturer's protocol. Pre-amplification was performed with PreAmp kit (Life Technologies) using 3 µl of input RNA for the adipose tissue and 6 µl for the serum. The profiling was performed using an Applied Biosystems 7900 HT real-time PCR instrument. MiR profiling was normalized using the median of the overall expression of the miRs on each array (ΔCt). Fold change was calculated based on the estimated mean difference ($2^{-\Delta\Delta Ct}$). Ct values less than or equal to 30 or a Ct 31 were established as cut-off in adipose tissue and serum, respectively, to obtain the best selection of miRs for validation phase using another technique (RT-qPCR) sensible to Ct value. Only miRs expressed in all the samples were selected for analyses and fold changes ≥ 1.9 and ≤ -1.9 were considered potentially significant.

Confirming the profiling results

For validation, single specific miRs expressed in both adipose tissue and serum samples across all the participants were selected from the profiling analysis assuming a possible tissue cross-talk. These miRs were miR-16-5p, miR-451a, miR-223-3p, miR-18a-5p, miR-19a-3p and miR-486-5p. In addition, miR-363 was validated owing to its extreme pattern of differential expression in adipose tissue between the studied groups. Samples from the premenopausal women (SAWEs, n=9) and postmenopausal women (miRBody, HRT: n=9, No HRT: n=12) were used for validation. Ten ng of adipose tissue RNA was transcribed to cDNA with the TaqMan MicroRNA Reverse Transcription Kit (Applied Biosystems, by Life Technologies) and the quantitative real-time PCR (RT-qPCR) was subsequently performed with TaqMan MicroRNA Assays using 1 μl of cDNA according to the manufacturer's protocol. The amplification program was performed with slight modifications: at 95°C for 20 s, 40 cycles at 95°C for 1 s and at 60°C for 20 s. The RNU44 value of each adipose sample was used for normalization. ΔCt values were calculated as $\Delta Ct=Ct_{miR}$ X-Ct_{RNU44}. Relative expressions were calculated by using the $2^{-\Delta Ct}$ method. For the serum, 5 μl of sample was used for cDNA synthesis with the TaqMan MicroRNA Reverse Transcription Kit and the RT-qPCR was subsequently performed using 1 μl of cDNA. The amplification program was performed as described above. Each reaction was performed in duplicate. The cel-miR-39 value was used for normalization. ΔCt values were calculated as $\Delta Ct=Ct_{miR\ X}$-$Ct_{cel\text{-}miR\text{-}39}$. Relative expressions were calculated by using the $2^{-\Delta Ct}$ method.

mRNA and protein analyses from adipose tissue

Based on the literature, only previously validated miR targets were analyzed. SAWEs samples from the premenopausal women (n=12) and mirBody samples from the postmenopausal women (HRT; n=9, No HRT; n=12) were used for the analyses. mRNA levels of different gene transcripts were assessed in adipose tissue starting from total RNA extracted as described above. RNA was treated with DNase using a TURBO DNA-free Kit (Ambion, by Life Technologies), then reverse transcribed with iScript cDNA Synthesis (BioRad, Hercules, California, U.S.A.). BRAF, ESR1, BCL2, CCND1, AKT1, were tested with specific primers (BioRad) in RT-qPCR with Sybr Green (iTaq Universal SYBR Green Supermix, BioRad). Data were normalized to the GAPDH expression level and reported as mean ± SD. The relative amount of each mRNA was calculated as $\Delta Ct = Ct_{mRNA} - Ct_{GAPDH}$ and relative quantification of mRNA expression was calculated with the $2^{-\Delta Ct}$ method.

Proteins from the subcutaneous adipose tissue samples (n=5/group) were lysed with Pierce RIPA Buffer (#89900, Thermo Scientific) including Halt Protease and phosphatase inhibitor cocktail (1x), EDTA 0.5M (1x) and Pepstatin A (1x). 50 μl of the mix was used per 10 mg of adipose tissue sample. Average sample size was 45 mg. TissueLyzer was used at 20 Hz for 2 min to homogenize the samples. After homogenization samples were centrifuged for 20 min at 12000 g at +4°C and supernatant with no lipid remains was collected. A Pierce™ BCA Protein Assay Kit (#23225, Thermo Scientific) was used for the protein concentration measurements according to the manufacturer's instructions with slight modifications (10 μl of 1/6 diluted sample was pipetted to each well).

For the western blot, 16 μg of protein was pipetted into each well of the gel (Criterion Precast TGX, 4-20%, #567-1094, Bio-Rad). Run time was 45 min at 200 V. The samples were blotted on nitrocellulose membrane for 2 h at 400 mA +4°C, after which the membrane was dyed with Ponceau and imaged (BioRad Universal Hood II, Biorad Laboratories, CA). Blocking was performed for 1 h on a swing with commercial Blocking Buffer (#1168-9670, Licor). The primary antibodies from Cell signaling were diluted 1:1000 (B-Raf Rabbit mAb D9T6S, #14815, BCL-2 Antibody Human Specific #2872, Cyclin D1 Antibody #2922, AKT1 C73H10 Rabbit # 2938S) and from Santa Cruz Technologies 1:200 (ESRalpha HC-20 sc-543) and 1:40000 (GAPDH) to blocking buffer and PBS-0.1% Tween 20 mix (1:2). Primary antibody was let to affect overnight at +4°C. Anti-Rabbit Odyssey IRDye anti-rabbit IgG (H+L) (1 mg/ml 926-32213) was used as a secondary antibody (1:20000) for 1 h covered on a swing. Imaging and quantitation were performed with Odyssei CLx (LI-COR) Image Studio Ver 2.0.38. All samples were loaded on the same gel and were normalized to Ponceau dye (marker at 60 kDa).

REFERENCES

1. Pöllänen E, Sipilä S, Alen M, Ronkainen PH, Ankarberg-Lindgren C, Puolakka J, Suominen H, Hämäläinen E, Turpeinen U, Konttinen YT, Kovanen V. Differential influence of peripheral and systemic sex steroids on skeletal muscle quality in pre- and postmenopausal women. Aging Cell. 2011; 10:650–60.
2. Kaprio J, Koskenvuo M. Genetic and environmental factors in complex diseases: the older Finnish Twin Cohort. Twin Res. 2002; 5:358–65.

3. Ronkainen PH, Kovanen V, Alén M, Pöllänen E, Palonen EM, Ankarberg-Lindgren C, Hämäläinen E, Turpeinen U, Kujala UM, Puolakka J, Kaprio J, Sipilä S. Postmenopausal hormone replacement therapy modifies skeletal muscle composition and function: a study with monozygotic twin pairs. J Appl Physiol (1985). 2009; 107:25–33.

Supplementary Table 1: Medical history, current medication and health characteristics of the pre- and postmenopausal women in both studies

	Pre SAWEs (n=17)	HRT SAWEs (n=5)	No HRT SAWEs (n=5)	HRT miRBody (n=16)	No HRT miRBody (n=17)
**Gynecological surgery:**					
Oophorectomy	-	(4)	-	1 (1)	(1)
Hysterectomy	-	(4)	-	3 (2)	6 (3)
Uterine/vaginal prolapse	-	-	-	3	2 (1)
Rectal prolapse	-	-	-	3	2
Myoma	-	-	-	3	1
Incontinence	-	-	-	1 (2)	4
Other operations	-	-	-	2	2
**Regular medications for:**					
Hypertension	-	3	3	6	4
Osteoarthritis	-	-	-	4	6
Hypercholesteromia	-	-	-	5	-
Hypothyroidism	-	-	-	1	2
Asthma	1	-	-	1	1
Depression	-	-	-	3	1
**Smoking (current daily)**	5	1	1	2	-
**Physical activity:**					
Sedentary	-			2	3
Moderately active	6	3	2	8	9
Active	11	2	3	5	5

Related to the Figure 1 and Table 1.
"-"means no use or no history.
Among miRBody women gynecological surgery indicates the operation in which the subcutaneous adipose tissue sample was obtained. Numbers in the brackets () stand for operations performed before these studies. Pre= premenopausal women, HRT= postmenopausal users of hormone replacement therapy, No HRT= postmenopausal non-users of hormone replacement therapy.

Supplementary Table 2: Fold changes (FC) of the profiled miRs in adipose tissue (A) and serum (B).

See Supplementary File 1

Supplementary Table 3: Numerical values for validated miR correlations illustrated in clustered heatmap

		miR-16-5p	miR-223-3p	miR-363-3p	miR-18a-5p	miR-19a-3p	miR-451a	miR-486-5p
**Spearman correlation**								
	**AGE**	0.582	0.474	0.487	0.463	0.381	0.503	0.477
	**BMI**	-0.095	0.185	-0.121	-0.217	-0.156	-0.248	-0.275
	**CRP**	0.017	0.094	0.158	-0.019	-0.046	-0.074	-0.149
	**E_2**	-0.409	-0.273	-0.488	-0.447	-0.372	-0.362	-0.400
	**FSH**	0.446	0.316	0.563	0.435	0.313	0.375	0.333
**P-value**								
	**AGE**	<0.001	0.005	0.004	0.007	0.029	0.003	0.005
	**BMI**	0.607	0.310	0.508	0.234	0.394	0.170	0.127
	**CRP**	0.926	0.604	0.38	0.914	0.798	0.681	0.408
	**E_2**	0.018	0.125	0.004	0.009	0.033	0.039	0.021
	**FSH**	0.009	0.073	0.001	0.011	0.077	0.032	0.059
**FDR (P<0.05)**								
	**AGE**	<0.001	0.007	0.007	0.008	0.029	0.007	0.007
	**BMI**	0.607	0.543	0.593	0.543	0.551	0.543	0.543
	**CRP**	0.926	0.926	0.926	0.926	0.926	0.926	0.926
	**E_2**	0.037	0.125	0.028	0.032	0.046	0.046	0.037
	**FSH**	0.026	0.077	0.007	0.026	0.077	0.056	0.077

Related to the Figure 4.
FDR: false discovery rate, BMI: body mass index, CRP: high sensitive C-reactive protein, E2: 17β-estradiol, FSH: follicle stimulating hormone.
