# Supplementary material for: Menopause and adipose tissue: miR-19a-3p is sensitive to hormonal replacement
Source: Oncotarget. 2017 Dec 18;9(2):2279–94. doi: 10.18632/oncotarget.23406 (PMC5788639; doi:10.18632/oncotarget.23406)
Supplement: Supplementary file 2 [file oncotarget-09-2279-s002.docx]

| **Table S2:** Fold changes (FC) of the profiled miRs in adipose tissue (A) and serum (B).    Pre= premenopausal women. HRT= postmenopausal users of hormone replacement therapy. No HRT= postmenopausal non-users of hormone replacement therapy. Related to the Figure 2. | | | | | | | | |
| --- | --- | --- | --- | --- | --- | --- | --- | --- |
| **A) Profiled miR/Adipose tissue** | **FC No HRT vs. Pre** | **FC HRT vs. Pre** | **FC HRT vs. No HRT** |  | **B) Profiled miR/Serum** | **FC No HRT vs. Pre** | **FC HRT vs. Pre** | **FC HRT vs. No HRT** |
| hsa-let-7a-4373169 | 0.87 | 0.66 | 0.76 |  | hsa-let-7d-4395394 | 0.63 | 0.81 | 1.27 |
| hsa-let-7c-4373167 | 1.01 | 0.53 | 0.52 |  | hsa-let-7e-4395517 | 1.05 | 1.91 | 1.82 |
| hsa-let-7d-4395394 | 1.47 | 1.22 | 0.83 |  | hsa-miR-15b-4373122 | 0.76 | 0.85 | 1.12 |
| hsa-let-7e-4395517 | 1.50 | 1.25 | 0.83 |  | hsa-miR-16-4373121 | 0.45 | 1.08 | 2.39 |
| hsa-let-7f-4373164 | 0.88 | 0.70 | 0.80 |  | hsa-miR-17-4395419 | 0.51 | 1.29 | 2.52 |
| hsa-let-7g-4395393 | 1.04 | 1.21 | 1.16 |  | hsa-miR-18a-4395533 | 0.10 | 0.24 | 2.36 |
| hsa-miR-10a-4373153 | 0.97 | 0.87 | 0.90 |  | hsa-miR-19a-4373099 | 0.34 | 0.90 | 2.63 |
| hsa-miR-10b-4395329 | 1.04 | 0.80 | 0.77 |  | hsa-miR-19b-4373098 | 0.63 | 1.06 | 1.68 |
| hsa-miR-15a-4373123 | 1.20 | 0.47 | 0.39 |  | hsa-miR-20a-4373286 | 0.52 | 1.38 | 2.65 |
| hsa-miR-15b-4373122 | 1.80 | 0.68 | 0.38 |  | hsa-miR-20b-4373263 | 0.51 | 1.34 | 2.62 |
| hsa-miR-16-4373121 | 2.49 | 1.29 | 0.52 |  | hsa-miR-21-4373090 | 0.59 | 1.25 | 2.13 |
| hsa-miR-17-4395419 | 1.57 | 0.75 | 0.48 |  | hsa-miR-24-4373072 | 0.83 | 1.15 | 1.39 |
| hsa-miR-18a-4395533 | 2.19 | 0.83 | 0.38 |  | hsa-miR-25-4373071 | 0.43 | 0.92 | 2.14 |
| hsa-miR-18b-4395328 | 3.20 | 1.05 | 0.33 |  | hsa-miR-26a-4395166 | 0.82 | 1.62 | 1.98 |
| hsa-miR-19a-4373099 | 1.93 | 1.25 | 0.65 |  | hsa-miR-27a-4373287 | 0.44 | 1.06 | 2.39 |
| hsa-miR-19b-4373098 | 1.46 | 0.77 | 0.53 |  | hsa-miR-28-3p-4395557 | 0.46 | 0.86 | 1.88 |
| hsa-miR-20a-4373286 | 1.20 | 0.66 | 0.55 |  | hsa-miR-30b-4373290 | 1.20 | 1.81 | 1.51 |
| hsa-miR-20b-4373263 | 1.63 | 0.80 | 0.49 |  | hsa-miR-30c-4373060 | 0.70 | 1.13 | 1.61 |
| hsa-miR-21-4373090 | 1.36 | 0.67 | 0.50 |  | hsa-miR-92a-4395169 | 0.71 | 1.14 | 1.61 |
| hsa-miR-22-4373079 | 1.27 | 1.08 | 0.85 |  | hsa-miR-93-4373302 | 0.37 | 1.30 | 3.55 |
| hsa-miR-23a-4373074 | 1.51 | 0.77 | 0.51 |  | hsa-miR-99b-4373007 | 1.07 | 2.50 | 2.32 |
| hsa-miR-24-4373072 | 1.34 | 0.65 | 0.48 |  | hsa-miR-106a-4395280 | 0.50 | 1.43 | 2.87 |
| hsa-miR-25-4373071 | 1.50 | 0.72 | 0.48 |  | hsa-miR-106b-4373155 | 0.42 | 0.80 | 1.93 |
| hsa-miR-26a-4395166 | 1.07 | 0.97 | 0.91 |  | hsa-miR-122-4395356 | 0.76 | 0.98 | 1.30 |
| hsa-miR-26b-4395167 | 1.02 | 0.62 | 0.61 |  | hsa-miR-126-4395339 | 0.81 | 1.72 | 2.12 |
| hsa-miR-27a-4373287 | 1.48 | 0.88 | 0.59 |  | hsa-miR-139-5p-4395400 | 1.29 | 3.53 | 2.74 |
| hsa-miR-27b-4373068 | 1.02 | 0.55 | 0.54 |  | hsa-miR-140-5p-4373374 | 0.65 | 1.38 | 2.12 |
| hsa-miR-28-3p-4395557 | 1.07 | 0.82 | 0.76 |  | hsa-miR-142-3p-4373136 | 0.63 | 1.05 | 1.68 |
| hsa-miR-28-5p-4373067 | 1.00 | 0.57 | 0.57 |  | hsa-miR-145-4395389 | 0.77 | 1.10 | 1.42 |
| hsa-miR-29a-4395223 | 1.37 | 0.95 | 0.69 |  | hsa-miR-146a-4373132 | 1.45 | 1.98 | 1.37 |
| hsa-miR-29b-4373288 | 1.47 | 0.66 | 0.45 |  | hsa-miR-146b-5p-4373178 | 0.73 | 0.93 | 1.26 |
| hsa-miR-29c-4395171 | 1.28 | 0.64 | 0.50 |  | hsa-miR-150-4373127 | 1.41 | 1.00 | 0.71 |
| hsa-miR-30b-4373290 | 1.31 | 1.01 | 0.77 |  | hsa-miR-185-4395382 | 0.13 | 0.44 | 3.28 |
| hsa-miR-30c-4373060 | 1.41 | 0.98 | 0.69 |  | hsa-miR-186-4395396 | 0.80 | 1.10 | 1.37 |
| hsa-miR-31-4395390 | 0.54 | 0.61 | 1.14 |  | hsa-miR-191-4395410 | 0.70 | 1.13 | 1.61 |
| hsa-miR-34a-4395168 | 1.55 | 1.18 | 0.76 |  | hsa-miR-192-4373108 | 0.49 | 0.82 | 1.70 |
| hsa-miR-92a-4395169 | 2.29 | 0.76 | 0.33 |  | hsa-miR-195-4373105 | 0.36 | 0.83 | 2.29 |
| hsa-miR-93-4373302 | 1.20 | 0.65 | 0.54 |  | hsa-miR-197-4373102 | 0.89 | 1.02 | 1.15 |
| hsa-miR-95-4373011 | 1.31 | 1.50 | 1.14 |  | hsa-miR-199a-3p-4395415 | 0.34 | 0.52 | 1.52 |
| hsa-miR-99a-4373008 | 1.58 | 0.97 | 0.61 |  | hsa-miR-221-4373077 | 0.57 | 0.93 | 1.61 |
| hsa-miR-99b-4373007 | 1.23 | 1.06 | 0.86 |  | hsa-miR-222-4395387 | 0.57 | 0.78 | 1.37 |
| hsa-miR-100-4373160 | 1.27 | 0.62 | 0.48 |  | hsa-miR-223-4395406 | 0.31 | 0.66 | 2.12 |
| hsa-miR-101-4395364 | 0.78 | 0.62 | 0.79 |  | hsa-miR-320-4395388 | 0.45 | 1.06 | 2.35 |
| hsa-miR-103-4373158 | 1.29 | 0.86 | 0.67 |  | hsa-miR-323-3p-4395338 | 3.02 | 3.37 | 1.12 |
| hsa-miR-106a-4395280 | 1.44 | 0.59 | 0.41 |  | hsa-miR-328-4373049 | 0.78 | 1.66 | 2.14 |
| hsa-miR-106b-4373155 | 1.35 | 0.80 | 0.59 |  | hsa-miR-331-3p-4373046 | 0.50 | 0.95 | 1.90 |
| hsa-miR-107-4373154 | 1.31 | 0.77 | 0.59 |  | hsa-let-7b-4395446 | 0.35 | 0.79 | 2.26 |
| hsa-miR-125a-5p-4395309 | 1.20 | 1.25 | 1.04 |  | hsa-miR-342-3p-4395371 | 1.43 | 0.95 | 0.67 |
| hsa-miR-125b-4373148 | 1.08 | 1.00 | 0.92 |  | hsa-miR-375-4373027 | 0.65 | 0.66 | 1.01 |
| hsa-miR-126-4395339 | 1.01 | 0.69 | 0.68 |  | hsa-miR-451-4373360 | 0.51 | 1.18 | 2.28 |
| hsa-miR-127-3p-4373147 | 0.79 | 0.67 | 0.85 |  | hsa-miR-484-4381032 | 0.55 | 1.06 | 1.92 |
| hsa-miR-128-4395327 | 1.12 | 0.65 | 0.58 |  | hsa-miR-486-5p-4378096 | 0.68 | 1.42 | 2.08 |
| hsa-miR-130a-4373145 | 1.77 | 0.91 | 0.51 |  | hsa-miR-574-3p-4395460 | 0.89 | 1.79 | 2.02 |
| hsa-miR-130b-4373144 | 1.14 | 0.77 | 0.68 |  | hsa-miR-660-4380925 | 0.38 | 0.53 | 1.41 |
| hsa-miR-132-4373143 | 0.98 | 0.85 | 0.87 |  |  |  |  |  |
| hsa-miR-133a-4395357 | 1.61 | 0.77 | 0.48 |  |  |  |  |  |
| hsa-miR-139-3p-4395424 | 1.02 | 0.53 | 0.52 |  |  |  |  |  |
| hsa-miR-139-5p-4395400 | 0.82 | 0.63 | 0.77 |  |  |  |  |  |
| hsa-miR-140-3p-4395345 | 1.29 | 0.87 | 0.67 |  |  |  |  |  |
| hsa-miR-140-5p-4373374 | 1.26 | 0.89 | 0.70 |  |  |  |  |  |
| hsa-miR-142-3p-4373136 | 2.54 | 1.34 | 0.53 |  |  |  |  |  |
| hsa-miR-143-4395360 | 1.27 | 0.83 | 0.65 |  |  |  |  |  |
| hsa-miR-145-4395389 | 1.34 | 0.74 | 0.55 |  |  |  |  |  |
| hsa-miR-146a-4373132 | 1.45 | 1.03 | 0.71 |  |  |  |  |  |
| hsa-miR-146b-5p-4373178 | 1.54 | 1.21 | 0.79 |  |  |  |  |  |
| hsa-miR-148a-4373130 | 1.37 | 1.15 | 0.84 |  |  |  |  |  |
| hsa-miR-149-4395366 | 1.36 | 1.57 | 1.16 |  |  |  |  |  |
| hsa-miR-150-4373127 | 1.16 | 0.88 | 0.77 |  |  |  |  |  |
| hsa-miR-152-4395170 | 0.86 | 0.64 | 0.74 |  |  |  |  |  |
| hsa-miR-181a-4373117 | 0.88 | 0.72 | 0.81 |  |  |  |  |  |
| hsa-miR-185-4395382 | 1.72 | 1.02 | 0.59 |  |  |  |  |  |
| hsa-miR-186-4395396 | 0.96 | 1.18 | 1.23 |  |  |  |  |  |
| hsa-miR-190-4373110 | 1.02 | 0.76 | 0.75 |  |  |  |  |  |
| hsa-miR-191-4395410 | 1.38 | 0.98 | 0.71 |  |  |  |  |  |
| hsa-miR-192-4373108 | 1.04 | 0.12 | 0.12 |  |  |  |  |  |
| hsa-miR-193a-3p-4395361 | 1.55 | 1.38 | 0.89 |  |  |  |  |  |
| hsa-miR-193a-5p-4395392 | 0.97 | 0.67 | 0.69 |  |  |  |  |  |
| hsa-miR-193b-4395478 | 1.18 | 1.03 | 0.88 |  |  |  |  |  |
| hsa-miR-194-4373106 | 0.96 | 0.51 | 0.54 |  |  |  |  |  |
| hsa-miR-195-4373105 | 1.08 | 0.76 | 0.71 |  |  |  |  |  |
| hsa-miR-196b-4395326 | 0.95 | 0.68 | 0.72 |  |  |  |  |  |
| hsa-miR-197-4373102 | 2.18 | 2.14 | 0.98 |  |  |  |  |  |
| hsa-miR-199a-5p-4373272 | 1.28 | 0.97 | 0.75 |  |  |  |  |  |
| hsa-miR-199a-3p-4395415 | 1.06 | 0.96 | 0.90 |  |  |  |  |  |
| hsa-miR-199b-5p-4373100 | 1.55 | 0.97 | 0.63 |  |  |  |  |  |
| hsa-miR-200c-4395411 | 0.78 | 0.96 | 1.23 |  |  |  |  |  |
| hsa-miR-204-4373094 | 1.20 | 0.80 | 0.66 |  |  |  |  |  |
| hsa-miR-210-4373089 | 1.76 | 1.18 | 0.67 |  |  |  |  |  |
| hsa-miR-214-4395417 | 1.11 | 0.84 | 0.76 |  |  |  |  |  |
| hsa-miR-215-4373084 | 0.97 | 0.52 | 0.54 |  |  |  |  |  |
| hsa-miR-218-4373081 | 1.48 | 0.75 | 0.50 |  |  |  |  |  |
| hsa-miR-221-4373077 | 1.81 | 1.31 | 0.72 |  |  |  |  |  |
| hsa-miR-222-4395387 | 1.63 | 1.30 | 0.80 |  |  |  |  |  |
| hsa-miR-223-4395406 | 1.73 | 0.77 | 0.44 |  |  |  |  |  |
| hsa-miR-224-4395210 | 1.02 | 0.85 | 0.83 |  |  |  |  |  |
| hsa-miR-301a-4373064 | 1.36 | 1.07 | 0.79 |  |  |  |  |  |
| hsa-miR-320-4395388 | 1.24 | 1.27 | 1.02 |  |  |  |  |  |
| hsa-miR-324-3p-4395272 | 0.88 | 0.45 | 0.51 |  |  |  |  |  |
| hsa-miR-324-5p-4373052 | 1.04 | 0.46 | 0.44 |  |  |  |  |  |
| hsa-miR-328-4373049 | 1.02 | 0.73 | 0.72 |  |  |  |  |  |
| hsa-miR-330-3p-4373047 | 1.48 | 0.65 | 0.44 |  |  |  |  |  |
| hsa-miR-331-3p-4373046 | 0.81 | 0.66 | 0.83 |  |  |  |  |  |
| hsa-miR-335-4373045 | 1.48 | 1.53 | 1.04 |  |  |  |  |  |
| hsa-miR-339-5p-4395368 | 0.72 | 0.65 | 0.89 |  |  |  |  |  |
| hsa-miR-340-4395369 | 1.19 | 1.09 | 0.92 |  |  |  |  |  |
| has-miR-155-4395459 | 1.21 | 0.78 | 0.64 |  |  |  |  |  |
| hsa-let-7b-4395446 | 1.38 | 1.17 | 0.85 |  |  |  |  |  |
| hsa-miR-342-3p-4395371 | 1.40 | 1.32 | 0.94 |  |  |  |  |  |
| hsa-miR-345-4395297 | 1.00 | 0.61 | 0.61 |  |  |  |  |  |
| hsa-miR-362-5p-4378092 | 0.63 | 0.75 | 1.19 |  |  |  |  |  |
| hsa-miR-363-4378090 | 1.34 | 0.01 | 0.01 |  |  |  |  |  |
| hsa-miR-365-4373194 | 0.84 | 1.51 | 1.80 |  |  |  |  |  |
| hsa-miR-370-4395386 | 0.69 | 0.41 | 0.60 |  |  |  |  |  |
| hsa-miR-374a-4373028 | 1.03 | 0.78 | 0.76 |  |  |  |  |  |
| hsa-miR-374b-4381045 | 1.37 | 1.05 | 0.77 |  |  |  |  |  |
| hsa-miR-376a-4373026 | 0.77 | 0.47 | 0.61 |  |  |  |  |  |
| hsa-miR-379-4373349 | 1.04 | 0.57 | 0.55 |  |  |  |  |  |
| hsa-miR-382-4373019 | 0.53 | 0.32 | 0.61 |  |  |  |  |  |
| hsa-miR-411-4381013 | 0.82 | 0.75 | 0.92 |  |  |  |  |  |
| hsa-miR-423-5p-4395451 | 1.17 | 0.96 | 0.82 |  |  |  |  |  |
| hsa-miR-425-4380926 | 1.45 | 0.80 | 0.55 |  |  |  |  |  |
| hsa-miR-451-4373360 | 3.74 | 0.49 | 0.13 |  |  |  |  |  |
| hsa-miR-452-4395440 | 1.36 | 1.41 | 1.04 |  |  |  |  |  |
| hsa-miR-454-4395434 | 1.17 | 0.68 | 0.58 |  |  |  |  |  |
| hsa-miR-455-3p-4395355 | 0.64 | 0.71 | 1.12 |  |  |  |  |  |
| hsa-miR-483-5p-4395449 | 0.86 | 0.79 | 0.92 |  |  |  |  |  |
| hsa-miR-484-4381032 | 1.01 | 0.77 | 0.76 |  |  |  |  |  |
| hsa-miR-486-5p-4378096 | 5.96 | 0.86 | 0.14 |  |  |  |  |  |
| hsa-miR-489-4395469 | 1.65 | 1.45 | 0.88 |  |  |  |  |  |
| hsa-miR-491-5p-4381053 | 0.92 | 1.00 | 1.09 |  |  |  |  |  |
| hsa-miR-500-4395539 | 1.29 | 1.34 | 1.04 |  |  |  |  |  |
| hsa-miR-501-5p-4373226 | 1.71 | 1.38 | 0.80 |  |  |  |  |  |
| hsa-miR-504-4395195 | 0.86 | 0.97 | 1.12 |  |  |  |  |  |
| hsa-miR-512-3p-4381034 | 0.15 | 0.30 | 1.98 |  |  |  |  |  |
| hsa-miR-517a-4395513 | 0.19 | 0.30 | 1.55 |  |  |  |  |  |
| hsa-miR-517c-4373264 | 0.24 | 0.36 | 1.53 |  |  |  |  |  |
| hsa-miR-532-3p-4395466 | 1.20 | 0.86 | 0.71 |  |  |  |  |  |
| hsa-miR-532-5p-4380928 | 1.25 | 0.84 | 0.67 |  |  |  |  |  |
| hsa-miR-539-4378103 | 0.84 | 0.48 | 0.58 |  |  |  |  |  |
| hsa-miR-574-3p-4395460 | 1.14 | 1.24 | 1.08 |  |  |  |  |  |
| hsa-miR-590-5p-4395176 | 0.90 | 0.71 | 0.79 |  |  |  |  |  |
| hsa-miR-628-5p-4395544 | 0.86 | 1.13 | 1.30 |  |  |  |  |  |
| hsa-miR-642-4380995 | 0.57 | 0.27 | 0.48 |  |  |  |  |  |
| hsa-miR-652-4395463 | 0.88 | 0.53 | 0.60 |  |  |  |  |  |
| hsa-miR-660-4380925 | 1.70 | 1.31 | 0.77 |  |  |  |  |  |
| hsa-miR-671-3p-4395433 | 0.87 | 0.99 | 1.14 |  |  |  |  |  |
| hsa-miR-708-4395452 | 1.48 | 1.29 | 0.87 |  |  |  |  |  |
| hsa-miR-744-4395435 | 0.90 | 0.63 | 0.70 |  |  |  |  |  |
| hsa-miR-874-4395379 | 0.49 | 0.42 | 0.86 |  |  |  |  |  |
| hsa-miR-885-5p-4395407 | 1.19 | 0.99 | 0.83 |  |  |  |  |  |
| hsa-miR-886-3p-4395305 | 1.58 | 1.63 | 1.03 |  |  |  |  |  |
| hsa-miR-886-5p-4395304 | 1.06 | 1.13 | 1.07 |  |  |  |  |  |
| hsa-miR-891a-4395302 | 3.42 | 3.50 | 1.02 |  |  |  |  |  |
| hsa-miR-211-4373088 | 0.72 | 0.99 | 1.37 |  |  |  |  |  |
| hsa-miR-212-4373087 | 1.07 | 1.22 | 1.13 |  |  |  |  |  |
| hsa-miR-376c-4395233 | 0.65 | 0.50 | 0.76 |  |  |  |  |  |
| hsa-miR-511-4373236 | 1.14 | 0.86 | 0.76 |  |  |  |  |  |
| hsa-miR-323-3p-4395338 | 0.68 | 0.56 | 0.81 |  |  |  |  |  |
| hsa-miR-339-3p-4395295 | 1.08 | 1.71 | 1.59 |  |  |  |  |  |
| hsa-miR-455-5p-4378098 | 0.89 | 0.78 | 0.88 |  |  |  |  |  |
| hsa-miR-494-4395476 | 1.18 | 1.01 | 0.86 |  |  |  |  |  |
| hsa-miR-134-4373299 | 1.02 | 1.90 | 1.85 |  |  |  |  |  |
| hsa-miR-299-5p-4373188 | 1.21 | 0.79 | 0.66 |  |  |  |  |  |
| hsa-miR-342-5p-4395258 | 0.87 | 1.04 | 1.19 |  |  |  |  |  |
| hsa-miR-433-4373205 | 0.71 | 0.49 | 0.69 |  |  |  |  |  |
| hsa-miR-505-4395200 | 0.91 | 0.60 | 0.66 |  |  |  |  |  |
| hsa-miR-519d-4395514 | 0.26 | 0.24 | 0.95 |  |  |  |  |  |
| hsa-miR-502-3p-4395194 | 1.19 | 0.76 | 0.64 |  |  |  |  |  |
